# Editorial: Modulating microglia to enhance neuroplasticity for restoring brain function after stroke

**DOI:** 10.3389/fncel.2023.1232437

**Published:** 2023-06-22

**Authors:** Zongjian Liu, Dirk M. Hermann, Egor Dzyubenko, Guodong Cao, Xiang Cao

**Affiliations:** ^1^Beijing Rehabilitation Hospital, Capital Medical University, Beijing, China; ^2^Department of Neurology, University Hospital Essen, University of Duisburg-Essen, Essen, Germany; ^3^Department of Neurology, University of Pittsburgh, Pittsburgh, PA, United States; ^4^Geriatric Research Education and Clinical Center, US Department of Veterans Affairs (VA) Pittsburgh Healthcare System, Pittsburgh, PA, United States; ^5^Department of Neurology, Nanjing Drum Tower Hospital, Affiliated Hospital of Medical School, Nanjing University, Nanjing, China

**Keywords:** microglia, neuroplasticity, stroke, neuroprotection, polarization, neuroinflammation

Despite recent improvements in recanalizing therapies (namely thrombolysis and mechanical thrombectomy) in the acute phase of ischemic stroke, stroke remains the leading cause of disability and a major cause of death worldwide (Hacke et al., 2008; Nogueira et al., 2018). Neuroprotection treatments aiming at the survival of injured neurons have without exception failed in the past 30 years (Savitz and Fisher, 2007). Neurorehabilitation currently is the only measure to enhance stroke outcome and to restore long-term function during the subacute and chronic phases in stroke patients (Richards and Cramer, 2023). The recovery potential of ischemic brain tissue is largely dictated by neuronal plasticity (Murphy and Corbett, 2009). Plasticity change include morphological and structural remodeling of axons, dendrites and synapses (Murphy and Corbett, 2009), which are largely mediated by glial cells, such as microglia and astrocytes (Dzyubenko and Hermann, 2023). Since spontaneous neuroplasticity-dependent functional recovery after stroke is mostly incomplete, the development of new treatment strategies aimed at enhancing plasticity and promoting neurological recovery are urgently required.

Microglia, the resident immune cells in the central nervous system, maintain the homeostasis of the brain. Microglia in the infarct core and peri-infarct zones are activated by damage-associated molecular patterns (Dzyubenko and Hermann, 2023), and then release cytokines that attract peripheral blood leukocytes, including neutrophil, monocytes and macrophages, into the injured brain (Shi et al., 2019). Microglia can be activated and polarize toward either pro-inflammatory or anti-inflammatory phenotypes in response to changes in the local CNS microenvironment (Yu et al., 2021). The anti-inflammatory microglia secret trophic factors to repair the damaged brain, whereas the pro-inflammatory microglia produce high concentrations of destructive mediators that may worsen ischemic brain injury. However, microglia-induced pro-inflammatory responses may still have beneficial effects on neuronal plasticity via synapse pruning, which affects neuronal network formation and function following ischemic stroke (Dzyubenko and Hermann, 2023). Neuronal network activation, in turn, modifies microglial polarization, as supported by the observations that transcranial magnetic stimulation induces the acquisition of an anti-inflammatory phenotype of microglia (Bai et al., 2023; Eichler et al., 2023). Hence, microglia are involved in neurorehabilitation in the stroke brain. To further elucidate these multiple actions, we selected the Research Topic “*Modulating microglia to enhance neuroplasticity for restoring brain function after stroke*.”

In this Research Topic, seven manuscripts were included, comprising original research and comprehensive reviews. The focus was on investigating the impact of microglia-mediated inflammatory responses and phagocytosis on neuroplasticity and myelin repair in various stroke models. These studies revealed that microglia have both positive and negative effects on different stages of ischemic or hemorrhagic brain injury.

Li, Hu et al. presented a study to investigate the relationship between early exercise intervention, brain-derived exosomes, microglial activation, and synaptic plasticity in ischemic stroke induced by middle cerebral artery occlusion (MCAO) in rats. The study revealed that exercise intervention increased body weight, reduced cerebral infarct volume and enhanced neurological function. Furthermore, the exosome infusion combined with exercise treatment showed even better therapeutic efficacy to improve the above parameters. The study also indicated that exosomes participate in the neural repair process caused by exercise via regulating synaptic plasticity through inhibiting excessive activation of microglia.

Shen et al. described the role of microglia and astrocytes in neurobehavioral changes and pathological properties in response to rat embolic stroke induced by fluorescent microsphere injection at sequential time points of 6 h and 1, 7, and 14 days after modeling. The study found that microglia and astrocytes were activated and gathered in the region of infarcts in response to ischemic events, accompanied by vascular destruction and upregulation of CX3CL1, a transmembrane chemokine that impacts the contact between microglia and neurons. The time-dependent alteration of neurons, microglia, and astrocytes may be useful in determining the optimal time window for the treatment of ischemic stroke.

Raffaele and Fumagalli summarized recent evidence describing the dynamics of microglia activation in the ischemic brain, with a focus on their contribution to myelin damage and repair. The study found that microglia promote oligodendrocyte precursor cell (OPC) recruitment toward the ischemic lesion and preserve myelin integrity early after stroke. However, OPC can also be induced to immunosenescence, impairing their capacity to exert pro-regenerative and neuroprotective functions at later time points. In addition, microglia-derived extracellular vesicles have a beneficial impact on post-stroke remyelination and functional recovery. The results collected in this review highlight the importance of supporting the pro-remyelination functions of microglial cells after stroke.

Wang X.-Y. et al. systematically reviewed the latest research on the inflammatory role of microglia in early brain injury caused by subarachnoid hemorrhage (SAH), with a particular focus on single-cell transcriptomics. By identifying transcriptional signatures, a better understanding of the role of microglia in resting and disease states can be gained. During SAH, microglia exert their protective effects by expressing neuroprotective proteins, including neuroglobin and heme oxygenase-1. Additionally, microglia upregulate the expression of anti-inflammatory cytokines and reduce the production of pro-inflammatory cytokines, which further reduces neuronal apoptosis and the degree of brain edema. The authors also found that microglia are involved in the development of brain edema, neuronal apoptosis, and blood–brain barrier disruption after SAH through signaling pathways mediated by receptors such as toll-like receptor 4, calcium-sensing receptor, and triggering receptor expressed on myeloid cells-1.

In another review article, Wang Y. et al. discussed the role and mechanisms of microglia-mediated neuroinflammation and neuroplasticity after ischemia, providing an overview of the recent progress in this field. Microglia exert various functions depending on the communication with other cellular brain partners after ischemic stroke, such as neurons, astrocytes and oligodendrocytes. Microglia can exist in pro-inflammatory states, contributing to secondary brain damage, or secrete anti-inflammatory cytokines and neurotrophic factors, facilitating recovery after stroke. Activated microglia are thus considered as a double-edged sword in neurological recovery after stroke.

Li, Wang et al. provided an overview of the molecular mechanism controlling microglia phagocytosis and the potential role of physical exercise in this process. They summarized recent knowledge of factors regulating microglial phagocytosis, including metabolic reprogramming, lipid metabolism and ligand and receptor binding. These findings provide new targets for the treatment of synaptic pruning deficiency and Aβ clearance disorder, in which microglial phagocytosis plays a crucial role. Physical exercise and certain drugs may exert neural remodeling effects through these mechanisms.

Zhang et al. systematically reviewed the vital regulatory roles of N6-methyladenosine (m6A) RNA modification in microglia-mediated inflammation and ischemic stroke. As a significant regulatory modification in crucial biological processes in eukaryotes, m6A methylation can participate in the activation and polarization of microglia/macrophages and could also play a regulatory role in microglia-induced inflammatory responses after stroke. Current investigations suggest that m6A modification may enable the identification of prospective targets for the treatment of stroke, leading to the development of promising m6A inhibitors or agonists for clinical use in the future.

It is crucial to develop an effective strategy to regulate the activation of microglia and polarize them into the most protective phenotype during the chronic recovery stage of stroke. Furthermore, while microglia are the primary immune cells and phagocytes in the central nervous system, recent studies have indicated that peripheral immune cells including neutrophil, macrophage, lymphocytes, natural killer and dendritic cells also participate in brain injury and repair after stroke. Future studies should focus on the role of peripheral immune cells in post-ischemic brain injury and neuroplasticity, and their interaction with brain intrinsic microglial immune responses.

## Author contributions

XC and ZL drafted the manuscript. DH, ED, and GC edited and revised the manuscript. All authors contributed to the article and approved the submitted version.
